# Systematic Analysis and Expression Profiles of the 4-Coumarate: CoA Ligase (4CL) Gene Family in Pomegranate (*Punica granatum* L.)

**DOI:** 10.3390/ijms23073509

**Published:** 2022-03-23

**Authors:** Yuying Wang, Linhui Guo, Yujie Zhao, Xueqing Zhao, Zhaohe Yuan

**Affiliations:** 1 Co-Innovation Center for Sustainable Forestry in Southern China, Nanjing Forestry University, Nanjing 210037, China; wangyuying@njfu.edu.cn (Y.W.); glhnl@njfu.edu.cn (L.G.); z1184985369@njfu.edu.cn (Y.Z.); zhaoxq402@163.com (X.Z.); 2 College of Forestry, Nanjing Forestry University, Nanjing 210037, China

**Keywords:** pomegranate, 4-Coumarate-CoA ligase (4CL), gene cloning, lignin, flavonoids, phylogenetic analysis

## Abstract

*4-Coumarate:CoA ligase* (*4CL*, EC6.2.1.12), located at the end of the phenylpropanoid metabolic pathway, regulates the metabolic direction of phenylpropanoid derivatives and plays a pivotal role in the biosynthesis of flavonoids, lignin, and other secondary metabolites. In order to understand the molecular characteristics and potential biological functions of the *4CL* gene family in the pomegranate, a bioinformatics analysis was carried out on the identified *4CLs*. In this study, 12 *Pg4CLs* were identified in the pomegranate genome, which contained two conserved amino acid domains: AMP-binding domain Box I (SSGTTGLPKGV) and Box II (GEICIRG). During the identification, it was found that *Pg4CL2* was missing Box II. The gene cloning and sequencing verified that this partial amino acid deletion was caused by genome sequencing and splicing errors, and the gene cloning results corrected the *Pg4CL2* sequence information in the ‘Taishanhong’ genome. According to the phylogenetic tree, *Pg4CLs* were divided into three subfamilies, and each subfamily had 1, 1, and 10 members, respectively. Analysis of cis-acting elements found that all the upstream sequences of *Pg4CLs* contained at least one phytohormone response element. An RNA-seq and protein interaction network analysis suggested that *Pg4CL5* was highly expressed in different tissues and may participate in lignin synthesis of pomegranate. The expression of *Pg4CL* in developing pomegranate fruits was analyzed by quantitative real-time PCR (qRT-PCR), and the expression level of *Pg4CL2* demonstrated a decreasing trend, similar to the trend of flavonoid content, indicating *Pg4CL2* may involve in flavonoid synthesis and pigment accumulation. *Pg4CL3*, *Pg4CL7*, *Pg4CL8*, and *Pg4CL10* were almost not expressed or lowly expressed, the expression level of *Pg4CL4* was higher in the later stage of fruit development, suggesting that *Pg4CL4* played a crucial role in fruit ripening. The expression levels of *4CL* genes were significantly different in various fruit development stages. The results laid the foundation for an in-depth analysis of pomegranate *4CL* gene functions.

## 1. Introduction

The emergence of lignin enables terrestrial plants to stand upright and transport water for a long distance. Flavonoids are a crucial class of secondary metabolites of plants [1]. These two metabolites participate in regulating various physiological functions and improving the adaptability to environmental stress [2,3]. The *4CL* gene can encode multiple enzymes and exhibit distinct substrate affinities that appear to coincide with different metabolic functions [4]. Different 4CL isozymes can selectively catalyze cinnamic acid, *p*-coumaric acid and other substances to produce corresponding CoA thioesters. Some CoA thioesters can be synthesized by cinnamoyl-CoA reductase (CCR), and cinnamyl alcohol dehydrogenase (CAD) catalyze the reaction into the specific pathway of lignin biosynthesis [5], or through chalcone synthase (CHS) and chalcone isomerase (CHI) to guide the reaction into the specific pathway of flavonoid biosynthesis [6]. Members of the *4CL* gene family have now been identified in several species, such as *Arabidopsis* [7], rice [8], cotton [9], *Eucalyptus grandis* [10], and pear [11]. The 4CL proteins all contain two amino acid conserved structural domains: Box I (SSGTTGLPKGV) and Box II (GEICIRG) [12]; Box I is the AMP-binding functional domain, and this region is not only conserved in 4CL but is also a conserved sequence typical to all acyl-activated enzymes, and can be directly involved in the catalytic reaction, whereas Box II is only highly conserved in 4CL and is not directly involved in the catalysis; it may be related to the spatial conformation of the enzyme [13].

Lavhale et al. [13] found that the 4CL family may evolve separately after monocots and eudicots were isolated by phylogenetic analysis of 4CL protein sequences in different species. The *4CL* family genes in eudicots are divided into two groups, type I and type II [8]. Type I 4CL members of eudicots, such as *Arabidopsis At4CL1*, *At4CL2*, *At4CL4*, and *E. grandis Egr4CL1*, play a crucial role in regulating lignin biosynthesis; Type II 4CL members are closely related to flavonoid biosynthesis, *At4CL3* and *Egr4CL2* participate in flavonoid biosynthesis [3,10,14]. In addition, some genes contain the same highly conserved AMP structural domain as 4CL, and are classified as *4CL-like* [7,15]. Although these genes are similar to 4CL in structure and can be successfully clustered with 4CL in phylogeny, their evolutionary relationships and functions are still unknown [16]. Recently, it has been reported that *4CL* and *4CL-like* genes are able to regulate various physiological functions and improve the ability to adapt to the environment [17].

The pomegranate (*Punica granatum*) is not only of considerable ornamental value but it is also a medicinal and food fruit [18,19]. Pomegranates are rich in antioxidants, such as flavonoids, which are extremely effective against tumors, diabetes, and other diseases [20,21,22,23]. According to the differences in seed hardness, pomegranates are classified into soft-seeded, semi-soft-seeded, and hard-seeded varieties, and it was found that the lignin content of the pericarp of pomegranate was positively correlated with seed hardness [18]. Soft-seeded pomegranates are highly edible and more popular with the market and consumers. The characters of soft-seeded and high flavonoid content will be a new target of pomegranate quality breeding in the future. The identification and evolutionary analysis of the 4CL gene family has been reported on in recent years, but research on the *4CL* gene family in the pomegranate has not been reported on. The completion of pomegranate genome sequencing and the publication of data [24,25,26] provides data support for pomegranate gene function research. This study aimed to identify *Pg4CLs* gene members in the pomegranate ‘Taishanhong’ genome by bioinformatics methods, analyze their phylogeny, gene structure, conserved motifs, cis-acting elements, protein interaction network and tissue expression specificity, and explore their potential roles in pomegranate growth and development with different tissues. The gene *Pg4CL2* (*Pg001327.1*) was cloned, the reason for Box II deletion was analyzed, and its function was further studied.

## 2. Results

### 2.1. Identification of Pg4CL Family Genes and Gene Cloning of Pg001327.1 

**Figure 1:**
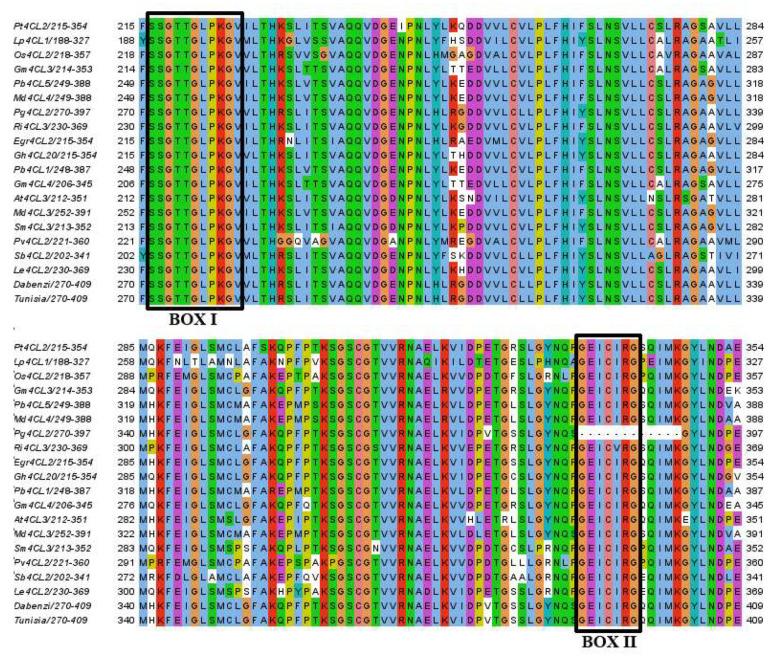
Multiple sequences alignment of *At4CL3* with other plant homologous proteins. Note: the prefixes Pt, Lp, Os, Gm, Pb, Md, Ri, Egr, Gh, At, Sm, Pv, Sb, and Le stand for *Populus tremuloides*, *Loblolly pine*, *Oryza Sativa*, *Glycine max*, *Pyrus bretschneideri*, *Malus domestica*, *Rubus idaeus*, *Eucalyptus grandis*, *Gossypium hirsutum*, *Arabidopsis thaliana*, *Salvia miltiorrhiza*, *Panicum virgatum*, *Sorghum bicolor*, and *Lithospermum erythrorhizon*, respectively. ‘Dabenzi’ and ‘Tunisia’ belong to pomegranate.

In this study, 16 candidate members were selected from the pomegranate genome of ‘Taishanhong’ by using the hidden Markov file of AMP binding domain (PF00501), and their AMP binding domain was verified by Pfam, NCBI, and SMART, respectively; the sequences of Box I and Box II without the 4CL amino acid conserved domain were eliminated. During the identification, it was found that although Box II of the *Pg001327.1* amino acid sequence was missing, *At4CL3* had the highest homology with *Pg001327.1* (61.79%) in the genome of ‘Taishanhong’. To explore whether Pg001327.1 belongs to the 4CL gene family, multiple alignments were performed between Pg001327.1, At4CL3, and homologous proteins of other species (FASTA S1, Figure 1), and only Box II of Pg001327.1 was deleted; Box I and Box II of amino acid sequences of other species were highly conserved. In order to explore the reasons for the deletion of the *Pg001327.1* amino acid sequence of Box II, a gene cloning experiment was carried out.

**Figure 2:**
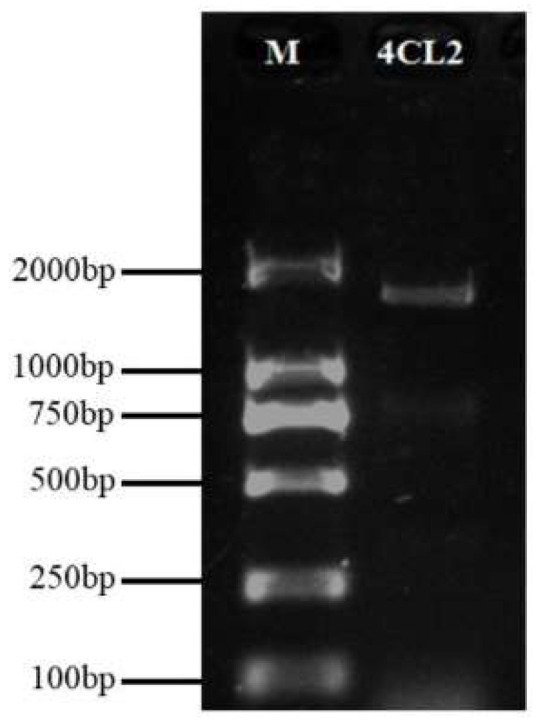
Detection of *Pg001327.1* PCR products by electrophoresis. Note: M: DL2000 DNA Marker; 4CL2: Target gene.

**Figure 3:**
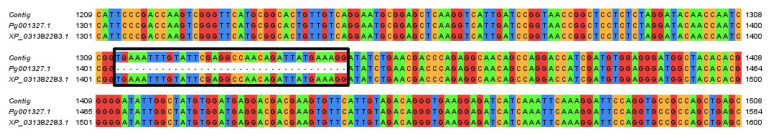
Multiple alignments of *Pg001327.1*, *XP_031382283.1* and sequencing results. This square represented that *Pg001327.1* was missing 36 nucleotides within positions 1404–1439 bp.

The *Pg001327.1* gene was amplified from the cDNA of ‘Taishanhong’ by PCR (Figure 2). The DNA was collected and sequenced after cloning. The total length of the PCR products was 1738 bp. The sequencing results were compared with pomegranate ‘Taishanhong’ genome sequence Pg001327.1 and ‘Tunisian’ genome sequence XP_ 031382283.1 (Figure 3), reaching 90.14% and 92.05%, respectively, with high homology. The Pg001327.1 gene was missing 36 nucleotides within positions 1404–1439 bp. Following the nucleotide positions of Pg001327.1 and XP_031382283.1, the Pg001327.1 nucleotide deletion sequence ‘GGTGAAATTTGTATTCGAGGCCAACAGATTATGAAAGG’ was translated, and the amino acid sequence was ‘GEICIRGQQIMK’, which contained Box II (GEICIRG), indicating that the deletion of amino acid sequence Box II of Pg001327.1 was caused by genome sequencing and assembly errors. According to the sequencing results, the sequence information of the genome Pg001327.1 was supplemented with missing nucleotides, and the corrected sequence was used for subsequent bioinformatics analysis.

### 2.2. Analysis of Physical and Chemical Properties

This study finally identified 12 candidate Pg4CLs genes in the pomegranate genome and named the Pg4CLs gene sequence as Pg4CL1–Pg4CL12 in the order of gene ID (Table 1). An analysis of the physical and chemical properties of Pg4CLs showed that the coding sequence of Pg4CLs was between 1629 and 2343 bp. In addition, the number of exons of Pg4CLs members was between 4 (Pg4CL8) and 14 (Pg4CL1), most of which were 6 exons. The Pg4CLs protein contains 542 (Pg4CL6) to 780 (Pg4CL1) amino acids, and the isoelectric point was between 5.49 (Pg4CL8) and 8.91 (Pg4CL12). A total of 58.3% of the Pg4CLs protein had a pI value greater than 7, which was slightly alkaline. The instability index of Pg4CLs protein ranged from 26.44 (Pg4CL6) to 49.09 (Pg4CL4), and 42% of Pg4CLs protein had good structural stability (less than 40 was stable). The total grand average of hydropathicity (GRAVY) of Pg4CL proteins ranged from −0.057 (Pg4CL4) to 0.159 (Pg4CL3). A total of 66.7% of Pg4CLs proteins were hydrophobic proteins (positive values were expressed as hydrophobic proteins). The analysis found that none of the Pg4CL proteins contained signal peptides and belonged to intracellular proteins. The prediction results of subcellular localization showed that most of the Pg4CL proteins (Pg4CL1, Pg4CL3, Pg4CL6, Pg4CL7, Pg4CL8, Pg4CL11, Pg4CL12) were localized in the plasma membrane, while the others were localized in the cytosol, extracellular, and peroxisome, respectively.

**Figure 4:**
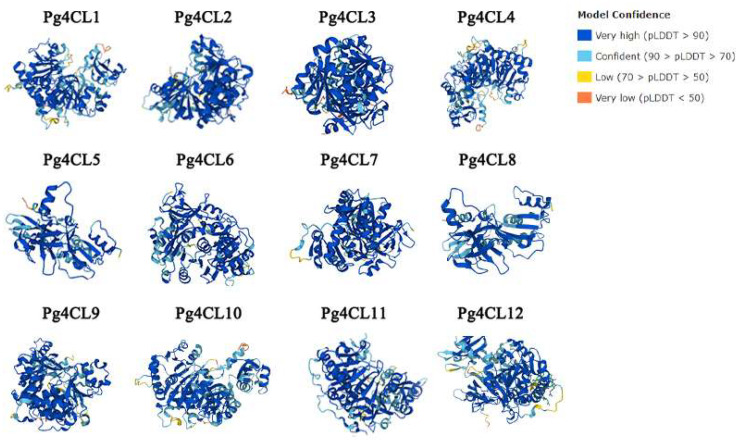
Tertiary structure of Pg4CL proteins. The tertiary structure of the protein was predicted through the AlphaFold. Model confidences are indicated by different colors. Blue, light blue, yellow, and red represent very high, confident, low, and very low confidence, respectively.

The prediction of the tertiary structure of proteins contributes to the study of their functions. AlphaFold produces a per-residue confidence score (pLDDT) between 0 and 100. The Pg4CL protein tertiary structure model had a very high confidence and great similarity (Figure 4).

### 2.3. Phylogenetic Analysis and Classification of the Pg4CL Gene Family

**Figure 5:**
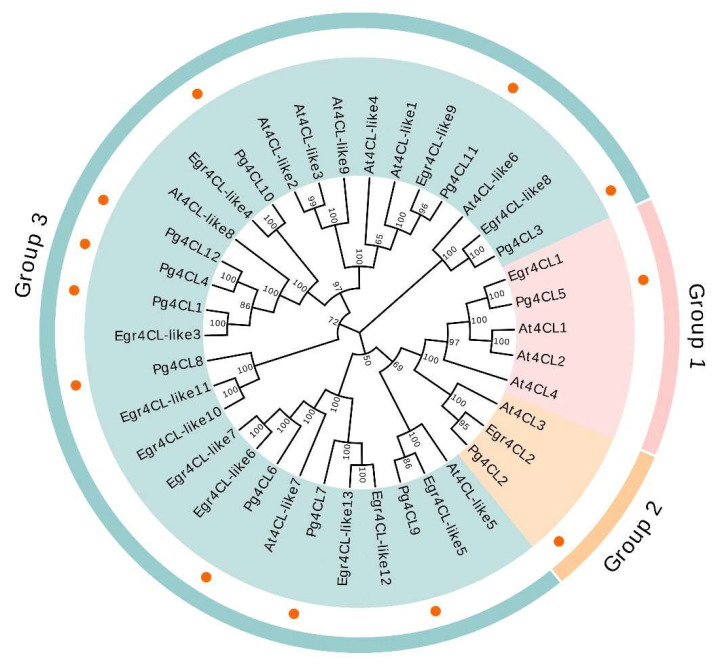
The phylogenetic tree of the 4CL gene family in *A. thaliana*, *E. grandis*, and *P. granatum.* The phylogenetic tree was constructed by using the neighbor-joining method with 1000 bootstrap replications. The prefixes At, Egr, and Pg stand for *A. thaliana*, *E. grandis*, *and P. granatum*.

In order to investigate the phylogenetic relationships of 4CL proteins, a phylogenetic tree was constructed using the neighbor-joining (NJ) method for a total of 38 amino acid sequences of 4CLs from *A. thaliana*, *E. grandis*, and *P. granatum* (Figure 5). The results showed that Pg4CL5 successfully clustered with At4CL1, At4CL2, At4CL4, and Egr4CL1, indicating that Pg4CL5 belonged to Group 1; Pg4CL2 clustered with At4CL3/Egr4CL2 and belonged to Group 2; the remaining 10 Pg4CLs clustered with At4CL-like/Egr4CL-like gene sequences and belonged to Group 3. Moreover, the functional studies of Group 1 and Group 2 genes in *A. thaliana* and *E. grandis* showed that they were involved in lignin synthesis and flavonoid synthesis. Therefore, it speculated that Pg4CL5 and Pg4CL2 play a pivotal role in lignin and flavonoid biosynthesis in the pomegranate.

### 2.4. Analysis of Conserved Motifs and Gene Structures of Pg4CL Gene Family 

**Figure 6:**
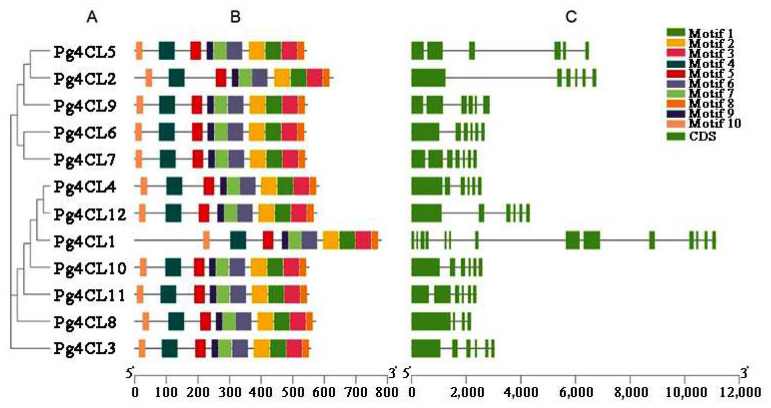
Phylogenetic tree, conservative protein motifs and gene structure of the Pg4CLs in pomegranate. (**A**) The phylogenetic tree of 12 Pg4CLs. (**B**) Conserved motifs in the 12 Pg4CLs. The motifs were identified by the MEME Suite. Different conserved motifs, numbers 1–10, are displayed in different colored boxes. (**C**) Gene structures of the 12 *Pg4CLs*.

A total of 10 conserved motifs were identified, numbered motifs 1–10 (Figure S1, Table 2). Pfam analysis showed that among these 10 motifs, five motifs (motif 1, motif 2, motif 4, motif 5 and motif 6) encoded AMP structural domains, of which motif 5 and motif 2 contained the conserved structural domains Box I (SSGTTGLPKGV) and Box II (GEICIRG), respectively. The conserved motif distribution of Pg4CLs protein was constructed based on the results of motif analysis (Figure 6B). The results showed that all Pg4CLs amino acid sequences contained all motifs, and the same subgroup contained similar motif distributions. The gene structure was visualized by TBtools (Figure 6C). The analysis showed that the length of the *Pg4CLs* gene was 2.1–12 kb. Among the 12 *Pg4CLs*, 9 of them consisted of 6 exons and 5 introns. In addition, *Pg4CL1* contained the most exons (14), resulting in a significantly longer gene length than other members. Group 1 and group 2 4CL members (*Pg4CL5*, *Pg4CL2*) had 6 exons. Because of their longer introns, their gene lengths were longer than that of members of group 3 (except for *Pg4CL1*). The number of introns ranged from 4 (*Pg4CL8*) to 14 (*Pg4CL1*).

### 2.5. Cis-Acting Elements in the Promoter Region of Pg4CLs 

**Figure 7:**
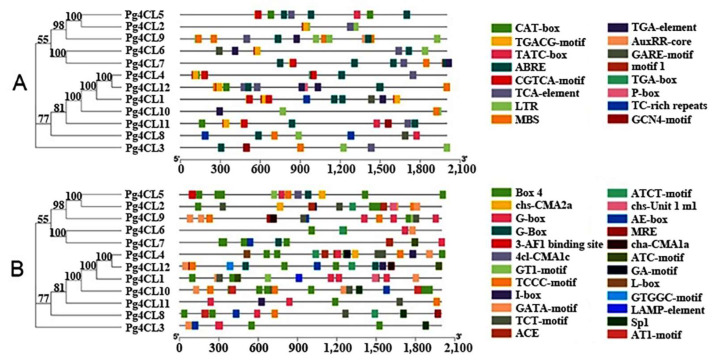
*Cis*-acting elments of *Pg4CLs*. (**A**) Hormone, stress, growth and development response elements of *Pg4CLs*; (**B**) Light response element of *Pg4CLs*. Note: abscisic acid response element (ABRE), growth hormone response elements (TGA-element, AuxRR-core, TGA-box), MeJA response elements (CGTCA-motif, TGACG-motif), gibberellin response elements (TATC-box, GARE-motif, P-box), and salicylic acid response elements (TCA-elements).

The cis-acting elements are the binding sites of transcription factors and play a crucial role in regulating gene expression function. Visualizing the identified cis-acting elements of *Pg4CLs*, all *Pg4CLs* contained at least one phytohormone response element (Figure 7A), including abscisic acid response element (ABRE), growth hormone response elements (TGA-element, AuxRR-core, TGA-box), MeJA response elements (CGTCA-motif, TGACG-motif), gibberellin response elements (TATC-box, GARE-motif, P-box), and salicylic acid response elements (TCA-elements). TGA-element was an auxin-responsive element and TGA-box was part of an auxin-responsive element. Among the 12 *Pg4CLs*, 8 contained MeJA response elements, 6 contained gibberellin response elements, 10 contained abscisic acid response elements, 9 contained salicylic acid response elements, and 7 contained auxin response elements. There were also low-temperature response elements (LTR) and drought response elements (MBS) in the promoter regions of some *Pg4CLs*, 6 contained LTR and 6 contained MBS. These *Pg4CLs* may respond to plant biotic and abiotic stresses. In addition, there was an MYB binding site (MBSI) involved in gene regulation of flavonoid biosynthesis in the promoter region of the *Pg4CL6*. Some *Pg4CLs* promoter regions also contained regulatory elements, such as endosperm expression (GCN4_motif) and meristem expression-related (CAT-box) (Figure 7A). These *Pg4CLs* may be closely related to plant growth and development. In addition, G-box and G-Box were cis-acting regulatory elements involved in light responsiveness. The binding sites of G-box and G-box transcription factors were different, the former was CACGTG and the latter was CACGTT. A total of 24 light-responsive elements appeared in this study (Figure 7B), of which the promoter regions of the *Pg4CLs* all contained a large number (5–17) of light-responsive elements, indicating that the *Pg4CLs* may be regulated by light. 

### 2.6. Protein Interaction Networks of Pg4CL Gene Family

**Figure 8:**
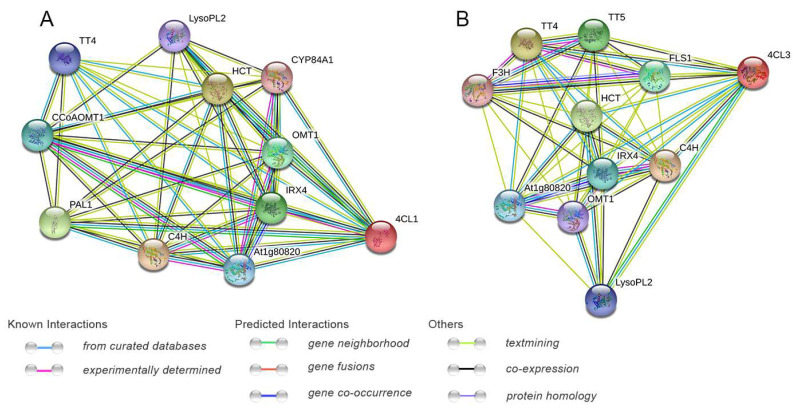
Function interaction network of Pg4CL5 (**A**)/Pg4CL2 (**B**) proteins. Note: TT4: chalcone synthase; CCoAOMT1: caffeoyl coenzyme A-O-methyltransferase; At1g80820: cinnamoyl-CoA reductase 2; C4H: cinnamate 4-hydroxylase; IRX4: cinnamoyl-CoA reductase 1; PAL1: phenylalanine ammonia-lyase 1; OMT1: flavone 3′-O-methyltransferase 1; HCT: shikimate O-hydroxycinnamoyltransferase; CYP84A1: ferulate 5-hydroxylase; LysoPL2: caffeoyl shikimate esterase; TT5: chalcone-flavanone isomerase; F3H: flavanone-3-hydroxylase; FLS1: flavonol synthase.

The protein interaction network prediction of 12 Pg4CLs showed no direct interaction between the proteins, which indicated that these proteins had no direct regulatory relationship and might play their functions. The potential functions of 4CL proteins Pg4CL5 and Pg4CL2 were predicted by protein interaction network analysis (Figure 8). The results are shown in Figure 8A,B, Pg4CL5 had high similarity with *A. thaliana* 4CL1 (*E*-value was 5.51 × 10^−234^), and 4CL1 was co-expressed with lignin synthesis proteins (C4H, IRX4, CYB84A1). Pg4CL2 was similar to *A. thaliana* 4CL3 (*E*-value was 3.9 × 10^−212^); 4CL3 was co-expressed with flavonoid synthesis proteins (TT4, TT5, OMT1, F3H, and FLS1). In addition, 4CL1 and 4CL3 were co-expressed with proteins in other metabolite synthesis pathways (At1g80820, HCT, LysoPL2).

### 2.7. Expression Analysis of Pg4CL Genes with RNA-Seq

**Figure 9:**
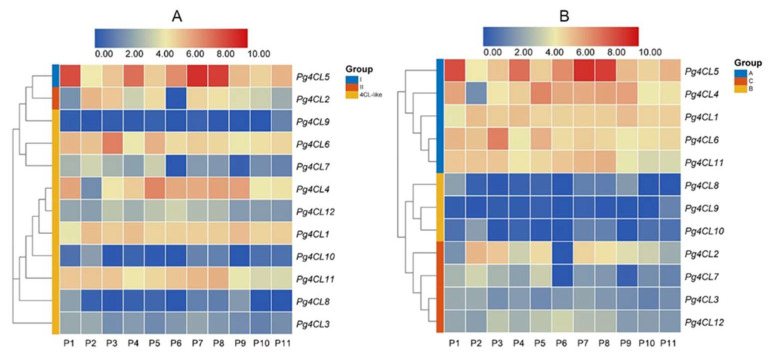
Heatmap of *Pg4CLs* gene expression in different tissues. Note: (**A**) Gene expression patterns of different subfamilies genes; (**B**) hierarchical clustering of gene expression patterns. P1: root; P2: leaf; P3: flower; P4, P7, P8: pericarp; P5: exocarp; P6, P9: pericarp; P10–P11: a mixture of flowers, leaves, fruits, and roots (cultivars P1–P6 are ‘Dabenzi’, cultivars P7, P8, P9, and P10 are ‘Tunisia’, ‘Baiyushizi’, ‘Wonderful’, ‘nana’ and ‘Black127’, respectively.

To investigate the expression pattern of Pg4CL genes in different tissues of pomegranate, the expression of Pg4CLs family genes was analyzed based on published transcriptome data of different tissues of the pomegranate. The results are shown in Figure 9, Pg4CL genes were expressed in leaves, roots, flowers, seed coats, and pericarps, but there were significant differences in the expression levels of genes in different subfamilies (Figure 9A). The class I 4CL gene (*Pg4CL5*) was highly expressed in different tissues of pomegranate, with the highest expression in the roots and pericarps. Class II 4CL gene (*Pg4CL2*) was highly expressed in the leaves, flowers, and exocarps of ‘Dabenzi’ and the pericarps of ‘Tunisia’ and ‘Baiyushizi’, but the lower expression in the roots and pericarps of ‘Dabenzi’. In addition, some of the 4CL-like genes (*Pg4CL1*, *Pg4CL4*, *Pg4CL6*, and *Pg4CL11*) were highly expressed in various tissues of pomegranate, but *Pg4CL8*, *Pg4CL9*, and *Pg4CL10* were lowly expressed or not expressed in various tissues, and the expression levels of 4CL-like genes were significantly different in various tissues. These results suggest that 4CL-like genes may be functionally differentiated.

Based on the differences in expression patterns, the Pg4CLs family was subjected to cluster analysis (Figure 9B), classified into three groups, A, B, and C. The gene expression level of group A was significantly higher than those of groups B and C and had higher expression in various tissues. The genes in group B were low or not expressed in all tissues, suggesting that these genes may have lost some of their functions during evolution. Genes in group C were expressed in leaves, flowers, and fruits, and the expression of different genes had some tissue specificity. Cluster analysis showed that different subfamily genes might have similar expression patterns, such as class I *Pg4CL5* and 4CL-like genes (*Pg4CL1*, *Pg4CL4*, *Pg4CL6*, *Pg4CL11*), which had high expression in pomegranate roots, leaves, flowers, seed coats, and pericarps.

### 2.8. qRT-PCR Analysis of Pg4CLs during Fruit Development in Pomegranate

**Figure 10:**
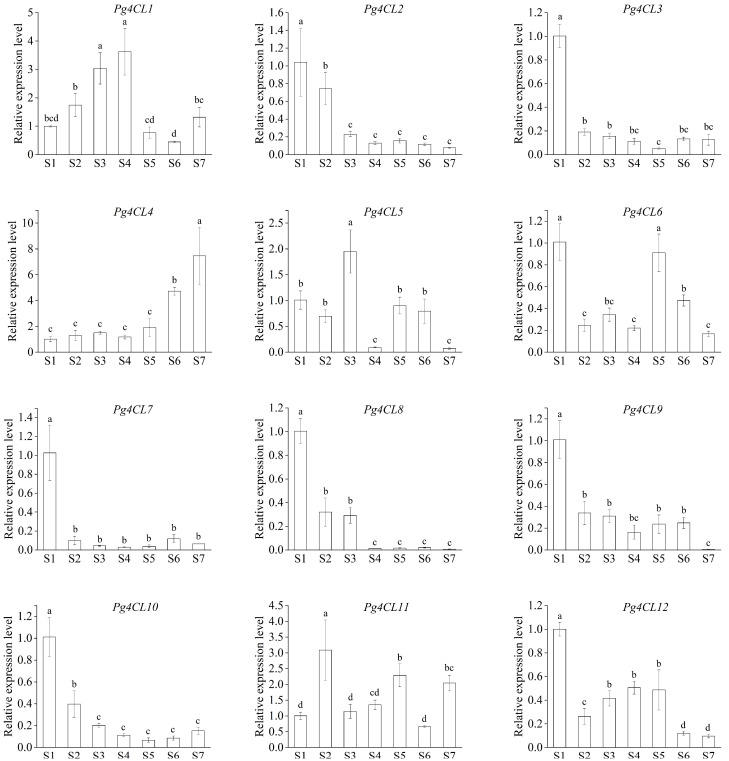
Relative expression levels of 12 *Pg4CL* from the pericarp during fruit development. Note: the seven stages of fruit development were S1–S7, and the specific dates of the seven stages were 15 July, 28 July, 10 August, 23 August, 5 September, 18 September, and 1 October, respectively. The vertical bar in the bar graph is standard error. Bars with different letters (a–d) indicate significant differences at *p* < 0.05 according to Duncan’s test.

To explore the putative function of *Pg4CLs* in fruit development, the expression patterns of *Pg4CLs* in pericarp of pomegranate were analyzed by qRT-PCR (Figure 10). The expression level of *Pg4CL1* showed an increasing trend in S1–S4 stages, the highest expression level was reached at the S4 stage. With the ripening of pomegranate fruit, the flavonoid content gradually decreased, and the expression level of *Pg4CL2* and flavonoid content demonstrated a similar trend, indicating that *Pg4CL2* was involved in the synthesis of flavonoids. The expression level of *Pg4CL4* was higher in the later stage of fruit development, suggesting that *Pg4CL4* played a crucial role in fruit ripening. The expression of *Pg4CL5* was higher in S5 stages. In addition, *Pg4CL3*, *Pg4CL7*, *Pg4CL8*, and *Pg4CL10* were almost not expressed or lowly expressed in S2–S7, and the expression levels in the S1 stage were significantly higher than those in S2–S7, and the expression levels of 4CL-like genes were significantly different in various fruit development stages.

## 3. Discussion

Multiple gene families encode 4CLs in higher plants, and the number of their gene members varies depending on the plant species. In recent years, the identification and functional analysis of 4CL gene families have been reported for many species, such as 13 members in *A. thaliana* [7], 13 members in *E. grandis* [10], 12 members in *P. bretschneideri* [11], 14 members in *O. sativa* [8], etc. In this study, the identification of the 4CL gene family members in the ‘Taishanhong’ genome revealed that *A. thaliana* At4CL3 had the highest amino acid sequence similarity to Pg001327.1. However, the At4CL3 in the pomegranate ‘Tunisia’ genome and other homologous protein sequence alignment results of different species showed that the Pg001327.1 was missing Box II, a conserved structural domain to the 4CL gene family. It was speculated that the Pg001327.1 might have the sequencing and splicing errors of the genome, which caused the deletion of Box II in the amino acid sequence. The Pg001327.1 gene cloning verified this conjecture, and the genome was corrected. A total of 12 Pg4CLs were identified in the genome of ‘Taishanhong’. Combined with the phylogenetic tree of 4CL amino acid sequences of *A. thaliana* and *E. grandis*, it was found that there was 2–4 4CL genes in class I, which was different from dicots plants [13]. In addition, only one gene was contained in the 4CL class I gene of the pomegranate and *E. grandis* [10]. By clustering with the model plant *A. thaliana*, the expression of its homologous gene *Pg4CL5* may be related to lignin synthesis based on the functions of *At4CL1*, *At4CL2*, and *At4CL5* [3,4]. Pomegranate, *E. grandis*, and *A. thaliana* contained only one 4CL class II gene, and its homologue *Pg4CL2* was presumed to be involved in flavonoid synthesis based on the function of *At4CL3* [3,4]. In addition, pomegranate contained 10 4CL-like genes, and although the functions of these 4CL-like genes were unknown, results from studies in other species suggested that both 4CL and 4CL-like genes can regulate various physiological functions of plants and improve their ability to adapt to their environment [17].

Based on the conserved motifs of Pg4CLs, it was found that all amino acid sequences of Pg4CLs contained 10 motifs, and similar motif distributions were found in the same subgroup. A gene structure analysis showed that Pg4CL genes contained 4–14 exons, including 6 exons in class I and class II 4CL genes, which was consistent with the number of exons in *A. thaliana* (3–6) and *Gossypium hirsutum* (4–6) [9,27], indicating that the structure of 4CL class I and class II genes were relatively conservative. However, the number of exons of 4CL-like varied greatly among different species, such as 1–5 in *A. thaliana* and 1–11 in *Populus pruinosa* [27]. This study showed that the members of Pg4CLs were conserved in both gene structure and protein conserved motifs.

*Cis*-acting elements existed in gene promoters and formed specific binding with transcription factors, which played a principal role in regulating the expression of target genes [28,29]. The promoter region of pomegranate Pg4CLs was enriched in a variety of cis-acting elements related to hormone responses and abiotic stresses, and this result was broadly consistent with the pear and *G. hirsutum* [9,30], indicating that 4CL gene promoter regulatory elements were somewhat conservative among different species. In addition, 66.7% of the promoter regions of Pg4CLs contained MYB transcription factor binding sites, among which, 6 Pg4CLs contained MYB binding sites (MBS) involved in drought induction, 4 Pg4CLs contained MBS involved in photoreaction, and there were also MBS involved in the regulation of flavonoid biosynthesis genes in the promoter region of Pg4CL6; these three MBS were found in the 4CL gene family of longan [31]. All Pg4CLs contained at least one phytohormone response element, such as the TGA-element, TATC-box, TGACG-motif, and TCA-elements, etc., which indicated that Pg4CLs might respond to auxin, gibberellin, jasmonic acid, salicylic acid, and other hormones involved in plant growth, development, and stress response regulation. In addition, 4CLs involved in stress resistance had been verified in plants, such as *Fraxinus mandshurica*, *G. hirsutum*, and poplar [9,17,27].

The protein interaction network prediction of 12 Pg4CLs revealed no interaction between their proteins, indicating no direct regulatory relationship between these proteins [13]. The interaction network analysis of Pg4CL5 and Pg4CL2 showed that Pg4CL5 was co-expressed with lignin biosynthesis, combined with a high expression of *Pg4CL5* in different tissues and the highest expression in roots and pericarps, indicating that *Pg4CL5* may be associated with lignin biosynthesis in various tissues. *Pg4CL5* had been cloned and validated in pomegranate ‘Baiyushizi’, and the conclusion is consistent with the present study [32]. In combination with the 4CL class II gene *Pg4CL2* co-expressed with flavonoid synthesis, the high expression of *Pg4CL2* in flowers, seed coats, and fruit may be associated with flavonoid synthesis and pigment accumulation [33]. In addition, it was also found that *Pg4CL2* had high expression in the pericarps of ‘Tunisia’ and ‘Baiyushizi’, but a lower expression in ‘Dabenzi’, which may be due to different genotypes and differences in gene expression patterns and may also be related to fruit characteristics [34]. We found that the expression of *Pg4CL2* decreased gradually, which was similar to the change trend of flavonoid content, indicating that *Pg4CL2* was involved in the synthesis of flavonoids. This is consistent with the results in *Pyrus bretschneideri* [30] and *Morus alba* [35]. To a large extent, the function of the 4CL-like gene is still unknown, and it has been reported that both 4CL and 4CL-like genes can regulate various physiological functions of plants. The expression of Pg4CLs family genes was analyzed based on published transcriptome data of different tissues of pomegranate, some 4CL-like genes (*Pg4CL1*, *Pg4CL6*, *Pg4CL11*) were highly expressed in pomegranate tissues, but some 4CL-like genes (*Pg4CL8*, *Pg4CL9*, *Pg4CL10*) were low or not expressed in various tissues. In addition, the expression of Pg4CL in developing pomegranate fruits was analyzed by quantitative real-time PCR (qRT-PCR), *Pg4CL1* showed an increasing trend in S1–S4 stages, the highest expression level was reached at the S4 stage; this is similar to the results in the Chinese pear [30]. *Pg4CL3*, *Pg4CL7*, *Pg4CL8*, and *Pg4CL10* were significantly increased, suggesting that these genes might play important roles in the early stage of fruit development [30]. The expression level of the 4CL-like gene in different fruit developments is different, which indicates that the function of the 4CL-like gene may be differentiated.

## 4. Materials and Methods

### 4.1. Genome and Transcriptome Data Sources

Pomegranate genome (‘Taishanhong’ ASM286412v1; ‘Tunisia’ ASM765513v2) [24,25], transcriptome data, and homologous amino acid sequences of At4CL3 in different species were downloaded from NCBI (http://www.ncbi.nlm.nih.gov/ (accessed on 4 November 2021)). The 4CL amino acid sequences of *E. grandis* and *Arabidopsis* were downloaded from the Phytozome database (https://phytozome.jgi.doe.gov/pz/portal.html (accessed on 4 November 2021)) and the TAIR database (https://www.arabidopsis.org (accessed on 4 November 2021)).

### 4.2. Identification of Pg4CL Family Genes

The Hidden Markov model (HMM) files corresponding to the AMP binding domain (PF00501) [9] were downloaded from the Pfam protein family database (http://pfam.xfam.org/ (accessed on 15 November 2021)) [36] and used as probes to screen the amino acid sequences of Pg4CLs (*E*-value ≤ 1.0 × 10^−10^). The protein structural domains were verified using Pfam, NCBI CDD, and SMART (http://smart.embl-heidelberg.de/ (accessed on 15 November 2021 )) [37,38], respectively, by removing repetitive sequences and sequences without AMP structural domains. Multiple sequence alignments were performed using MAFFT (Suita, Japan) [39] to identify whether they contained the conserved structural domains Box I (SSGTTGLPKGV) and Box II (GEICIRG).

### 4.3. Gene Cloning of Pg001327.1

Total RNA was extracted using the BioTeke plant total RNA extraction kit (centrifugal column type) (BioTeke Corporation Co., Ltd., Beijing, China), and cDNA was obtained by the reverse transcription kit (PrimeScript^TM^ RT reagent Kit with gDNA Eraser, TaKaRa Biomedical Technology Co., Ltd., Tokyo, Japan). Oligo 7 software (Cascade, CA, USA) was used to design cloning primers. Used the cDNA as a template, the upstream and downstream primers (F: 5′-ATGATATCTGTTGCCCCTTCT-3′, R: 5′TTATAAGGGAGTGGAGGAGG-3′) were used for PCR amplification. The PCR reaction system was 50 μL, including 25 μL 2 × Rapid Taq Master Mix (Vazyme Biotech Co., Ltd., Nanjing, China), 2 μL upstream and downstream primers, 2 μL cDNA template, and 21 μL ddH_2_O. The PCRs were performed on the Applied Biosystems 7500 (Thermo Fisher Scientific Inc., Waltham, MA, USA). The amplification procedures were 95 °C for 3 min; 95 °C for 15 s, 58 °C for 45 s, 72 °C for 1 min, a total of 35 cycles, and 72 °C for 5 min. The similarity between sequencing results and sequences Pg001327.1 and XP_031382283.1 was analyzed using BioXM 2.6 software, and the conservativeness of sequences was analyzed using Jalview software (Dundee, UK) after multiple sequence alignments using MAFFT [39].

### 4.4. Virtual Prediction of Physical and Chemical Properties

The ExPASy (https://web.expasy.org/protparam/ (accessed on 15 January 2022)) [40] was used to predict the physical and chemical properties of Pg4CL proteins, mainly including amino acid numbers, isoelectric point, instability index, grand average of hydropathicity (GRAVY). In addition, the SignalP 5.0 server (http://www.cbs.dtu.dk/services/SignalP (accessed on 15 January 2022)) was used to predict the signal peptide of the Pg4CL proteins [41]. Subcellular localization of the Pg4CL proteins was predicted using the Plant-mPLoc server (http://www.csbio.sjtu.edu.cn/bioinf/plant-multi/ (accessed on 19 January 2022)) [42]. Tertiary structures of Pg4CL proteins were predicted using the AlphaFold (https://www.alphafold.ebi.ac.uk/ (accessed on 19 January 2022)), which is based on a massive database and innovative algorithm [43].

### 4.5. Multiple Sequences Alignment and Phylogenetic Analysis 

Multiple sequences alignment was performed by MAFFT [39] using a total of 38 full-length protein sequences, including *Arabidopsis thaliana* (At, 13 sequences), *Punica granatum* (Pg, 12 sequences), and *Eucalyptus grandis* (Egr, 13 sequences). To investigate the phylogenetic relationships of the 4CL proteins, MEGA7.0 software [44] was used to construct a phylogenetic tree using the NJ method, Bootstrap 1000 repeats, and the others were set as default parameters. The online software EvolView (https://evolgenius.info/ (accessed on 11 February 2022)) was used to beautify the evolutionary tree [45]. According to the 4CL classification of *Arabidopsis* and *E. grandis*, Pg4CLs were classified and predicted.

### 4.6. Analysis of Gene Structure and Protein Conserved Motifs

According to the obtained pomegranate 4CL protein sequences and gene sequences (including introns, exons and upstream and downstream sequences), the online software GSDS 2.0 (http://gsds.gao-lab.org/ (accessed on 15 February 2022)) was used to analyze its gene structure [46]. The protein conserved motifs of Pg4CLs were analyzed online by MEME (http://meme-suite.org/tools/meme (accessed on 15 February 2022)) with a maximum number of motifs of 10 [47]. The phylogenetic tree of Pg4CLs, the obtained motifs, and the gene structure, were visualized using TBtools (Guangdong, China) [48].

### 4.7. Analysis of Cis-Acting Elements and Protein–Protein Interaction Networks

The promoter region contains conserved sequences required for specific binding and transcription initiation of RNA polymerase, which is generally located 1500–2000 bp upstream of the gene. The 2000 bp upstream base sequence of Pg4CL genes were extracted from the pomegranate genome database using TBtools [48]. The promoter characteristics and cis-acting elements of *Pg4CLs* were analyzed by the online website PlantCARE (http://bioinformatics.psb.ugent.be/webtools/plantcare/html/ (accessed on 15 February 2022)) [49], and the predicted results were sorted and simplified. Finally, TBtools was used to visualize cis-acting elements. The co-expressions of Pg4CLs, Pg4CL5, and Pg4CL2 in the pomegranate were analyzed (*A. thaliana* was selected as the model species) by using String (https://string-db.org (accessed on 15 February 2022)) [50]. 

### 4.8. RNA-Seq Analysis of 4CL Gene Family in Pomegranate 

RNA-Seq data of six pomegranate varieties (‘Dabenzi’, ‘Tunisia’, ‘Baiyushizi’, ‘Wonderful’, ‘Nana’, and ‘Black127’) were downloaded from the NCBI database, including root, leaf, flower, pericarp, exocarp, peel, and mixed samples of roots, leaf, flower, and fruit, as shown in Table 3. The TPM value was calculated by Kallisto v0.44.0 (CA, USA) [51], and different transcriptome data were homogenized by Log_2_ (TPM + 1), and then the expression heat map was drawn by TBtools [48].

### 4.9. Expression Patterns by Quantitative Real-Time PCR (qRT-PCR)

We collected samples of seven stages S1, S2, S3, S4, S5, S6, and S7 of fruit development in the pomegranate orchard of Baimashi Village, Tai’an City, Shandong Province. The specific dates of the seven stages were 15 July, 28 July, 10 August, 23 August, 5 September, 18 September, and 1 October. RNA was extracted from the exocarp of the samples, and cDNA was further obtained. Specific primers of Pg4CL2 and Pg4CL5 were designed (Table 4). The pomegranate PgActin was used as the internal reference gene. There were three biological replicates for each treatment. The qRT-PCR reaction system was 20 μL, including 10 μL Hieff^®^ qPCR SYBR Green Master Mix (Low Rox Plus) (Yeasen Biotechnology Co.,Ltd., Shanghai, China), 0.4 μL upstream and downstream primers, 2 μL cDNA template, and 7.2 μL ddH2O. The amplification procedures were 95 °C for 5 min; 95 °C for 15 s, 58 °C for 45 s, a total of 40 cycles. The relative expression level was analyzed by 2^−ΔΔCT^ method. The data were analyzed and plotted using SPSS 23.0 (Santa Clara, CA, USA) and Origin 2018 software (Newton, MA, USA), respectively.

## 5. Conclusions

In this study, 12 Pg4CLs were identified in the pomegranate genome, all of which contained AMP binding domains. According to the established 4CL classification of *A. thaliana* and *E. grandis*, Pg4CLs were divided into three subfamilies, I, II, and 4CL-like. Each subfamily had 1, 1, and 10 members, respectively. Box I and box II were highly conserved in class I and II members and relatively conserved in 4CL-like members. Among them, 4CL class I gene *Pg4CL5* was involved in lignin synthesis, and class II *Pg4CL2* was related to flavonoid synthesis. The results of gene cloning corrected the sequence information of *Pg4CL2* in the ‘Taishanhong’ genome and laid a foundation for its functional research.

## Figures and Tables

**Table 1:** The basic information of the Pg4CLs gene family in the pomegranate.

Gene Name	Gene ID	Location	CDS (bp)	Exon No.	AA	pI	Instability Index	GRAVY	Signal Peptide	Protein Localization
*Pg4CL1*	*Pg000024.1*	scaffold1:6623488:6634629	2343	14	780	8.81	47.22	0.059	No	Plasma membrane
*Pg4CL2*	*Pg001327.1*	scaffold10:76276:83034	1887	6	628	5.83	46.64	0.066	No	Cytosol
*Pg4CL3*	*Pg004702.1*	scaffold124:253916:256949	1674	6	557	6.55	39.93	0.159	No	Plasma membrane
*Pg4CL4*	*Pg005072.1*	scaffold13:4695163:4697720	1755	6	584	8.09	49.09	−0.057	No	Extracellular
*Pg4CL5*	*Pg007092.1*	scaffold15:2592574:2599061	1635	6	544	5.65	36.60	−0.034	No	Extracellular
*Pg4CL6*	*Pg007674.1*	scaffold152:355399:358070	1629	6	542	8.64	26.44	0.044	No	Plasma membrane
*Pg4CL7*	*Pg013255.1*	scaffold22:4058452:4060831	1638	7	545	7.65	38.71	−0.023	No	Plasma membrane
*Pg4CL8*	*Pg015184.1*	scaffold27:1309358:1311530	1722	4	573	5.49	40.47	0.024	No	Plasma membrane
*Pg4CL9*	*Pg016048.1*	scaffold3:3588368:3591221	1644	6	547	5.64	35.48	0.120	No	Cytosol
*Pg4CL10*	*Pg016524.1*	scaffold3:3410537:3413121	1659	6	552	8.45	42.89	0.153	No	Peroxisome
*Pg4CL11*	*Pg018200.1*	scaffold35:541618:543985	1656	6	551	8.7	41.33	0.037	No	Plasma membrane
*Pg4CL12*	*Pg029185.1*	scaffold85:64794:69120	1731	6	576	8.91	44.18	−0.010	No	Plasma membrane

**Table 2:** Information of the 10 motifs of Pg4CLs proteins.

Motif Code	Sequences	E_Value	Characteristic Domain
Motif 1	DGWLHTGDLCYFDDEGFLFIVDRJKELIKYKGYQVAPAELEALLLSHPEI	6.2 × 10^−341^	AMP-binding enzyme
Motif 2	GRLVPNMEAKIVDPETGAALPPNQSGELWLRGPTIMKGYLNBPEATAETI	5.20 × 10^−242^	AMP-binding enzyme
Motif 3	DAAVIPYPDEEAGEIPMAFVVRKPGSSITEEDVIDFVAKQVAPYKKIRRV	7.40 × 10^−236^	No
Motif 4	KGDVVLILLPNSJHFPIIYLSILSLGAVITTANPLSTPSEIAKQVRDSKP	9.60 × 10^−162^	AMP-binding enzyme
Motif 5	VRVDQDDTAAJLYSSGTTGTSKGVVLTHRNLIA	1.90 × 10^−160^	AMP-binding enzyme
Motif 6	KSSLTDKYDLSSLRQVGSGAAPLGKEVAEQFRKKFPHVELLQGYGMTEST	2.90 × 10^−160^	AMP-binding enzyme
Motif 7	LRAGETVVVMQKFEFEAMLRAIEKYRVTYLPVVPPIILALV	4.60 × 10^−102^	No
Motif 8	FIESIPKSPAGKILRRELIKK	2.30 × 10^−81^	No
Motif 9	NVFLCVLPLFHIYGLAVIILG	2.70 × 10^−53^	No
Motif 10	SGFCSKTGIFHSLRPPVPLPP	3.90 × 10^−32^	No

**Table 3:** RNA-Seq data of pomegranate.

NO.	Accession NO.	Cultivars	Sample Type	Library	Platform	Reference
P1	SRR5279396	‘Dabenzi’	root	Paired end	Illumina HiSeq 4000	[26]
P2	SRR5279397	‘Dabenzi’	leaf	Paired end	Illumina HiSeq 4000	[26]
P3	SRR5279395	‘Dabenzi’	flower	Paired end	Illumina HiSeq 4000	[26]
P4	SRR5279391	‘Dabenzi’	Pericarp	Paired end	Illumina HiSeq 4000	[26]
P5	SRR5279388	‘Dabenzi’	Exocarp	Paired end	Illumina HiSeq 4000	[26]
P6	SRR5279394	‘Dabenzi’	Pericarp	Paired end	Illumina HiSeq 4000	[26]
P7	SRR5678820	‘Tunisia’	Pericarp	Paired end	Illumina HiSeq 4000	[26]
P8	SRR5678819	‘Baiyushizi’	Pericarp	Paired end	Illumina HiSeq 4000	[26]
P9	SRR080723	‘Wonderful’	Pericarp	Paired end	Illumina HiSeq 2000	[52]
P10	SRR1055290	‘Nana’	Mixed samples of the root,	Single end	454 GS FLX Titanium	[53]
			leaves, flowers, and fruit			
P11	SRR1054190	‘Black127’	Mixed samples of the root,	Single end	454 GS FLX Titanium	[53]
			leaves, flowers, and fruit			

**Table 4:** Primers used in this study.

Gene	Primer Sequence (5′-3′)
*Pg4CL1*	F: GGATGGCTGGTTGAGGACAG; R: GCAGCATCTGCAATTTCGGG
*Pg4CL2*	F: CAGTGCTCTCGATGTGCCTA; R: TCATAATCTGTTGGCCTCGAA
*Pg4CL3*	F: AGAATGTGGGGAGGTCCCAG; R: CCTGCTGGAGACTTCGGTATC
*Pg4CL4*	F: AGAAGCAGGACAGATCCCGA; R: CTTCCCTGCCGGAGATTTCG
*Pg4CL5*	F: GCATTCGTCATCCGATCCAA; R: CGGATGCCAATTTCGCTCT
*Pg4CL6*	F: GCTTTCAGATTGCTCCTGCG; R: ATTCTGCACGTCCTCTTCGG
*Pg4CL7*	F: CCGGAGACCTCGGGTATTTC; R: TCGGGAAACGGAATGACCAC
*Pg4CL8*	F: AGTTGTACCGGTCGAGGATG; R: CTTGTAAGGCGCAACCTGAG
*Pg4CL9*	F: CGAATTGTGTGTCCGAAGCC; R: ACGACAAAGACGTCCCCATC
*Pg4CL10*	F: TACTCCCATCCCGACATTGC; R: GTCGTTTCACCACGAAAGCC
*Pg4CL11*	F: TCCGAACGGGAGATCTTTGC; R: TCGGGAAACGGGATAACAGC
*Pg4CL12*	F: GCAGATGCCGCTGTTATTCC; R: TTCCCTGCCGGAGATTTCG
*PgActin*	F: AGTCCTCTTCCAGCCATCTC; R: CACTGAGCACAATGTTTCCA

## Data Availability

The data presented in this study are available upon request from the corresponding author and the public pomegranate transcriptomes presented in this study are available in the insert article.

## Supplementary Materials

The following supporting information can be downloaded at: https://www.mdpi.com/article/10.3390/ijms23073509/s1.
