# Supplementary figures and images for: Systematic Analysis and Expression Profiles of the 4-Coumarate: CoA Ligase (4CL) Gene Family in Pomegranate (Punica granatum L.)
Source: Int J Mol Sci. 2022 Mar 23;23(7):3509. doi: 10.3390/ijms23073509 (PMC8999076; doi:10.3390/ijms23073509)

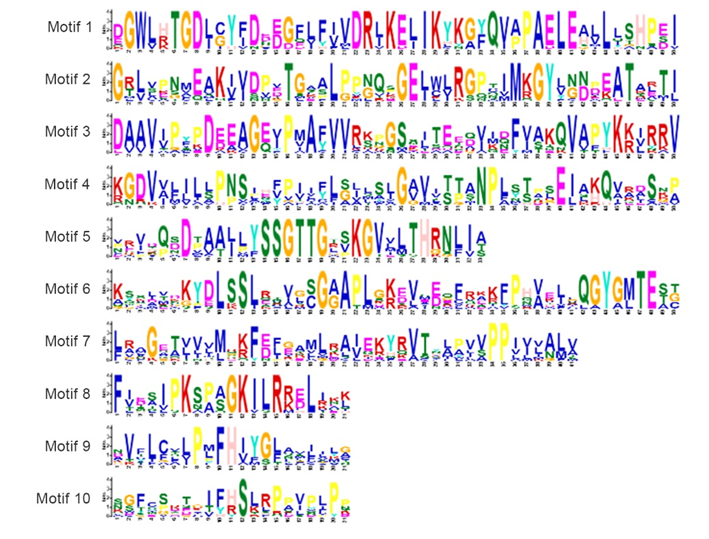

Supplement: Supplementary file 1 [file ijms-23-03509-s001.zip › Fig. S1. Conserved motifs of Pg4CLs protein in pomegranate.jpg]
